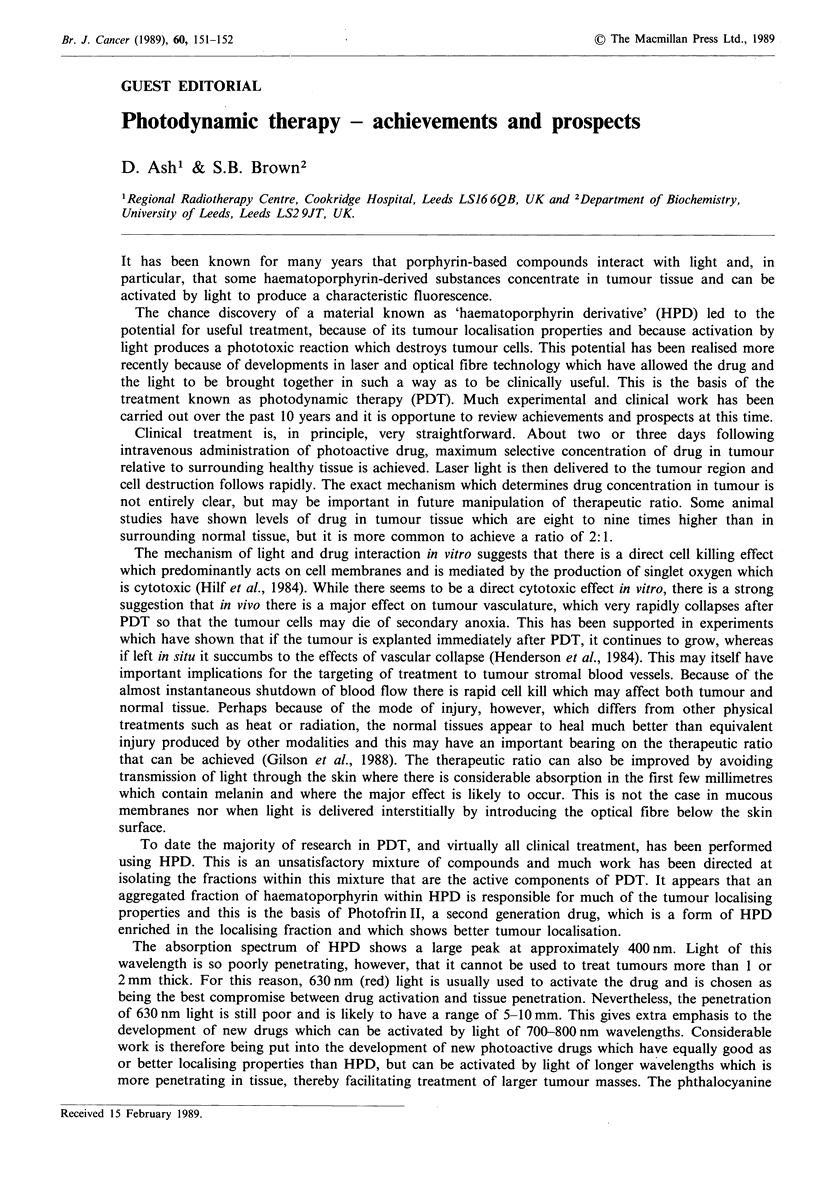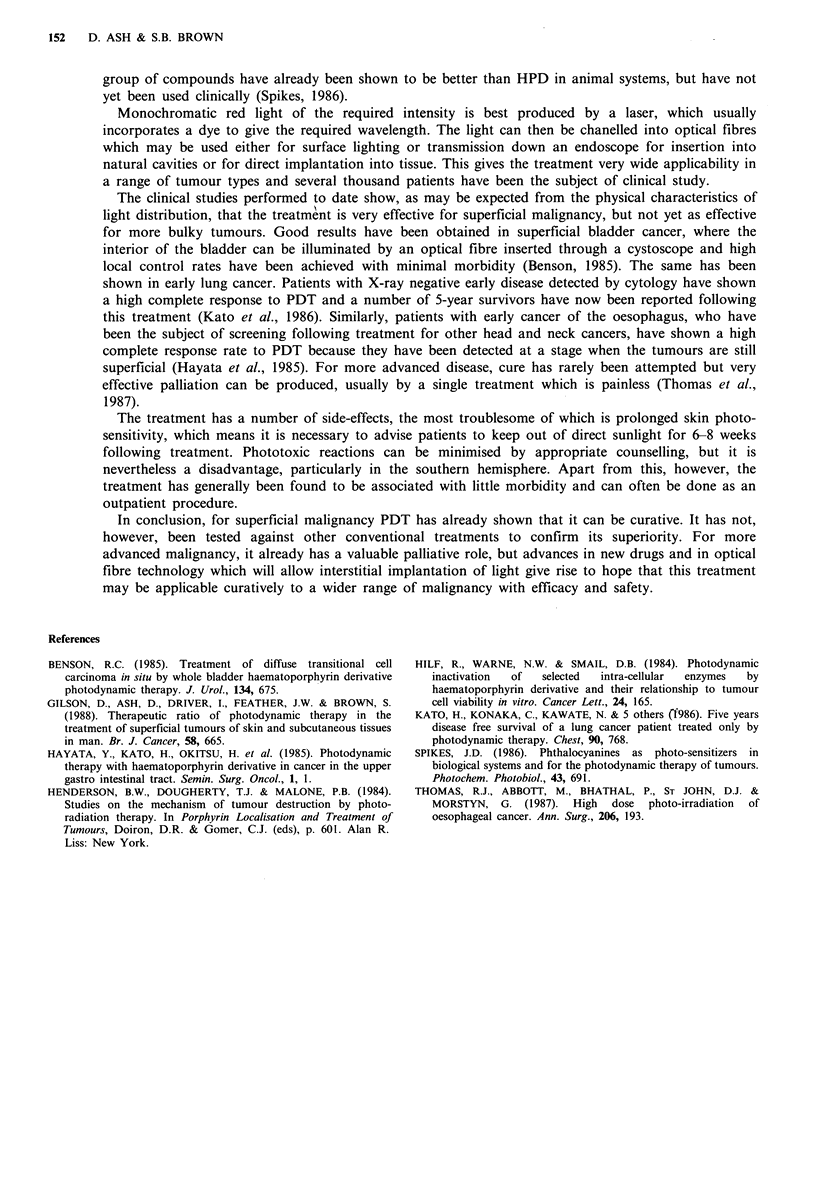# Photodynamic therapy--achievements and prospects.

**DOI:** 10.1038/bjc.1989.239

**Published:** 1989-08

**Authors:** D. Ash, S. B. Brown

**Affiliations:** Regional Radiotherapy Centre, Cookridge Hospital, Leeds, UK.


Br. J. Cancer (1989), 60, 151–152 © The Macmillan Press Ltd., 1989

GUEST EDITORIAL

### Photodynamic therapy – achievements and prospects

D. Ash[1] & S.B. Brown[2]

[1]*Regional Radiotherapy Centre, Cookridge Hospital, Leeds LS16 6QB, UK and* [2]*Department of Biochemistry, University of Leeds, Leeds LS2 9JT, UK.*

It has been known for many years that porphyrin-based compounds interact with light and, in particular, that some haematoporphyrin-derived substances concentrate in tumour tissue and can be activated by light to produce a characteristic fluorescence.

The chance discovery of a material known as 'haematoporphyrin derivative' (HPD) led to the potential for useful treatment, because of its tumour localisation properties and because activation by light produces a phototoxic reaction which destroys tumour cells. This potential has been realised more recently because of developments in laser and optical fibre technology which have allowed the drug and the light to be brought together in such a way as to be clinically useful. This is the basis of the treatment known as photodynamic therapy (PDT). Much experimental and clinical work has been carried out over the past 10 years and it is opportune to review achievements and prospects at this time.

Clinical treatment is, in principle, very straightforward. About two or three days following intravenous administration of photoactive drug, maximum selective concentration of drug in tumour relative to surrounding healthy tissue is achieved. Laser light is then delivered to the tumour region and cell destruction follows rapidly. The exact mechanism which determines drug concentration in tumour is not entirely clear, but may be important in future manipulation of therapeutic ratio. Some animal studies have shown levels of drug in tumour tissue which are eight to nine times higher than in surrounding normal tissue, but it is more common to achieve a ratio of 2:1.

The mechanism of light and drug interaction *in vitro* suggests that there is a direct cell killing effect which predominantly acts on cell membranes and is mediated by the production of singlet oxygen which is cytotoxic (Hilf *et al.*, 1984). While there seems to be a direct cytotoxic effect *in vitro*, there is a strong suggestion that *in vivo* there is a major effect on tumour vasculature, which very rapidly collapses after PDT so that the tumour cells may die of secondary anoxia. This has been supported in experiments which have shown that if the tumour is explanted immediately after PDT, it continues to grow, whereas if left *in situ* it succumbs to the effects of vascular collapse (Henderson *et al.*, 1984). This may itself have important implications for the targeting of treatment to tumour stromal blood vessels. Because of the almost instantaneous shutdown of blood flow there is rapid cell kill which may affect both tumour and normal tissue. Perhaps because of the mode of injury, however, which differs from other physical treatments such as heat or radiation, the normal tissues appear to heal much better than equivalent injury produced by other modalities and this may have an important bearing on the therapeutic ratio that can be achieved (Gilson *et al.*, 1988). The therapeutic ratio can also be improved by avoiding transmission of light through the skin where there is considerable absorption in the first few millimetres which contain melanin and where the major effect is likely to occur. This is not the case in mucous membranes nor when light is delivered interstitially by introducing the optical fibre below the skin surface.

To date the majority of research in PDT, and virtually all clinical treatment, has been performed using HPD. This is an unsatisfactory mixture of compounds and much work has been directed at isolating the fractions within this mixture that are the active components of PDT. It appears that an aggregated fraction of haematoporphyrin within HPD is responsible for much of the tumour localising properties and this is the basis of Photofrin II, a second generation drug, which is a form of HPD enriched in the localising fraction and which shows better tumour localisation.

The absorption spectrum of HPD shows a large peak at approximately 400 nm. Light of this wavelength is so poorly penetrating, however, that it cannot be used to treat tumours more than 1 or 2 mm thick. For this reason, 630 nm (red) light is usually used to activate the drug and is chosen as being the best compromise between drug activation and tissue penetration. Nevertheless, the penetration of 630 nm light is still poor and is likely to have a range of 5–10 mm. This gives extra emphasis to the development of new drugs which can be activated by light of 700–800 nm wavelengths. Considerable work is therefore being put into the development of new photoactive drugs which have equally good as or better localising properties than HPD, but can be activated by light of longer wavelengths which is more penetrating in tissue, thereby facilitating treatment of larger tumour masses. The phthalocyanine

Received 15 February 1989.

group of compounds have already been shown to be better than HPD in animal systems, but have not yet been used clinically (Spikes, 1986).

Monochromatic red light of the required intensity is best produced by a laser, which usually incorporates a dye to give the required wavelength. The light can then be chanelled into optical fibres which may be used either for surface lighting or transmission down an endoscope for insertion into natural cavities or for direct implantation into tissue. This gives the treatment very wide applicability in a range of tumour types and several thousand patients have been the subject of clinical study.

The clinical studies performed to date show, as may be expected from the physical characteristics of light distribution, that the treatment is very effective for superficial malignancy, but not yet as effective for more bulky tumours. Good results have been obtained in superficial bladder cancer, where the interior of the bladder can be illuminated by an optical fibre inserted through a cystoscope and high local control rates have been achieved with minimal morbidity (Benson, 1985). The same has been shown in early lung cancer. Patients with X-ray negative early disease detected by cytology have shown a high complete response to PDT and a number of 5-year survivors have now been reported following this treatment (Kato *et al.*, 1986). Similarly, patients with early cancer of the oesophagus, who have been the subject of screening following treatment for other head and neck cancers, have shown a high complete response rate to PDT because they have been detected at a stage when the tumours are still superficial (Hayata *et al.*, 1985). For more advanced disease, cure has rarely been attempted but very effective palliation can be produced, usually by a single treatment which is painless (Thomas *et al.*, 1987).

The treatment has a number of side-effects, the most troublesome of which is prolonged skin photosensitivity, which means it is necessary to advise patients to keep out of direct sunlight for 6–8 weeks following treatment. Phototoxic reactions can be minimised by appropriate counselling, but it is nevertheless a disadvantage, particularly in the southern hemisphere. Apart from this, however, the treatment has generally been found to be associated with little morbidity and can often be done as an outpatient procedure.

In conclusion, for superficial malignancy PDT has already shown that it can be curative. It has not, however, been tested against other conventional treatments to confirm its superiority. For more advanced malignancy, it already has a valuable palliative role, but advances in new drugs and in optical fibre technology which will allow interstitial implantation of light give rise to hope that this treatment may be applicable curatively to a wider range of malignancy with efficacy and safety.

### References

BENSON, R.C. (1985). Treatment of diffuse transitional cell carcinoma *in situ* by whole bladder haematoporphyrin derivative photodynamic therapy. *J. Urol.*, **134**, 675.

GILSON, D., ASH, D., DRIVER, I., FEATHER, J.W. & BROWN, S. (1988). Therapeutic ratio of photodynamic therapy in the treatment of superficial tumours of skin and subcutaneous tissues in man. *Br. J. Cancer*, **58**, 665.

HAYATA, Y., KATO, H., OKITSU, H. *et al.* (1985). Photodynamic therapy with haematoporphyrin derivative in cancer in the upper gastro intestinal tract. *Semin. Surg. Oncol.*, **1**, 1.

HENDERSON, B.W., DOUGHERTY, T.J. & MALONE, P.B. (1984). Studies on the mechanism of tumour destruction by photoradiation therapy. In *Porphyrin Localisation and Treatment of Tumours*, Doiron, D.R. & Gomer, C.J. (eds), p. 601. Alan R. Liss: New York.

HILF, R., WARNE, N.W. & SMAIL, D.B. (1984). Photodynamic inactivation of selected intra-cellular enzymes by haematoporphyrin derivative and their relationship to tumour cell viability *in vitro*. *Cancer Lett.*, **24**, 165.

KATO, H., KONAKA, C., KAWATE, N. & 5 others (1986). Five years disease free survival of a lung cancer patient treated only by photodynamic therapy. *Chest*, **90**, 768.

SPIKES, J.D. (1986). Phthalocyanines as photo-sensitizers in biological systems and for the photodynamic therapy of tumours. *Photochem. Photobiol.*, **43**, 691.

THOMAS, R.J., ABBOTT, M., BHATHAL, P., ST JOHN, D.J. & MORSTYN, G. (1987). High dose photo-irradiation of oesophageal cancer. *Ann. Surg.*, **206**, 193.